# Targeting Drug-Tolerant Persister Cancer Cells: Can Nanomaterial-Based Strategies Be Helpful for Anti-DTP Therapies?

**DOI:** 10.3390/pharmaceutics17111428

**Published:** 2025-11-04

**Authors:** Prachi Ghoderao, Eliza Kwiatkowska-Borowczyk, Sanjay Sahare, Hanna Dams-Kozlowska

**Affiliations:** 1 Department of Cancer Immunology, Poznan University of Medical Sciences, 8 Rokietnicka St., 60-806 Poznan, Poland; 2 Department of Diagnostics and Cancer Immunology, Greater Poland Cancer Centre, 15 Garbary St., 61-866 Poznan, Poland; eliza.kwiatkowska@wco.pl; 3 Faculty of Physics and Astronomy, Adam Mickiewicz University, 61-614 Poznan, Poland; sanjay.sahare@amu.edu.pl

**Keywords:** drug-tolerant persister cancer cells, therapeutic strategies, nanomaterial application

## Abstract

Therapeutic resistance remains a critical barrier in oncology, frequently leading to cancer relapse after initial treatment response. Growing evidence suggests the presence of drug-tolerant persisters (DTPs), a rare subpopulation of cancer cells that survives chemotherapy by entering a reversible specific adaptation. Unlike classical cell resistance, the DTP phenotype is independent of genetic changes and maintained through dynamic regulatory mechanisms. DTPs are phenotypically heterogeneous and can exhibit stem-like and quiescent cell phenotypes, non- or slow proliferation, and remarkable plasticity due to a di-pause-like state and executing epithelial–mesenchymal transition (EMT) or transdifferentiation processes. Despite advances in research, the molecular mechanisms underlying DTPs’ biology and their role in cancer relapse remain only partially understood. The review summarizes the current progress in processes that lead to the acquisition of cellular persistence status, which, in turn, constitute areas of vulnerability that can be exploited in cancer therapy. We highlight anti-DTP therapeutic strategies, including epigenetic modification, cell signaling and transcriptional regulation, metabolic reprogramming, and modification of cell interactions within the tumor microenvironment. Furthermore, we focus on the potential role of nanomaterials in the combat against DTPs. Nanoparticles not only act as part of the drug delivery process, enabling precise DTP targeting and enhancing intracellular drug accumulation, but their intrinsic properties can also be used to eradicate DTPs directly or by enhancing the effectiveness of other therapeutic strategies. The integrated approach offers strong potential to eliminate tumor persistence, prevent recurrence, and improve long-term patient outcomes beyond conventional therapies.

## 1. Introduction

Despite advancements in developing new anticancer therapies, drug resistance remains a significant challenge in cancer treatment. After various treatments, a small subset of cancer cells, known as minimal residual disease, may persist at the primary site, in the bloodstream, or at metastatic sites [1,2,3]. Drug resistance can occur due to a variety of mechanisms, such as increased drug inactivation, drug efflux from cancer cells, enhanced repair of chemotherapy-induced damage, activation of pro-survival pathways, and inactivation of cell death pathways [4]. These mechanisms are believed to reflect the existence of a small cell population within the tumor, genetically altered to confer resistance, that is selected during drug treatment. More recent studies revealed non-mutational mechanisms of drug resistance that may be intrinsic to the cancer cells or facilitated by their microenvironment. These include growth arrest or dormancy, phenotypic plasticity, metabolic shift, the role of the microenvironment, and the immune system.

Residual cancer cells are frequently compared to bacterial persisters. They comprise a tiny part of the bacterial population that survives lethal doses of antibiotics. Bacterial persisters have been found and described well in species like *Escherichia coli*, *Pseudomonas aeruginosa*, *Mycobacterium tuberculosis*, and *Salmonella enterica* subsp. enterica serovar Typhimurium and *Staphylococcus aureus* [5,6,7]. It is widely accepted that bacterial persisters are slow-growing or growth-arrested, and dormancy is one of the key mechanisms of persister formation [8,9]. Since antibiotics are primarily designed to target pathways active in growing bacteria, dormancy makes persisters particularly resistant to antibiotic therapy [10].

Acquired resistance to cancer drugs may involve a reversible “drug-tolerant” state. “Drug-tolerant cells” emerge for the first time from the research of Sharma et al., who modelled the acute response to various anti-cancer agents in drug-sensitive human tumor cell lines, including EGFR mutant NSCLC-derived cell line PC9 [11]. They observed that a small fraction, accounting for 0.3% of the original population, survived under drug concentrations exceeding the IC50 by 100-fold. These slowly cycling, quiescent cells were termed drug-tolerant persisters (DTPs). DTPs maintain viability via engagement of IGF-1 receptor signaling and an altered chromatin state that requires the histone demethylase RBP2/KDM5A/Jarid1A. Propagated in drug-free media, DTPs resume growth and rapidly reacquire drug sensitivity, like bacterial persisters. Drug-tolerant persisters have been reported in many other in vitro cancer models, including lung cancer [12,13], melanoma [14], breast cancer [15], and ovarian cancer [13].

By definition, DTP cells are cancer cells that survive prolonged exposure to anti-cancer drugs and are characterized by a reversible, non-genetically driven quiescent state. Terms such as “tolerant,” “dormant,” “quiescent,” and “senescent” have been used to describe rare tumor cell populations that withstand chronic drug treatment. However, while these terms overlap, the relationship between these alternative cell states and the DTP cell phenotype remains unclear. The DTP state is viewed as a temporary and reversible phase, enabling a small subset of tumor cells to survive initial anticancer treatment until more permanent resistance mechanisms develop [16,17,18].

It is important that DTPs are generated through specific interaction with the administered drug in a given microenvironment and do not solely arise from the intrinsic properties of specific cells. There is no consensus when it comes to the origin of the DTPs; however, understanding how persister cells arise may be crucial to developing adequate treatments for MDR (Multi-Drug Resistance) and preventing disease relapse. There are studies indicating that DTPs evolve from rare pre-existing cells, with different characteristics depending on the tumor being studied. Preexisting cells with high levels of aldehyde dehydrogenase (ALDH) were observed in a gastric cell line [19], whereas high levels of histone demethylases, tyrosine kinase AXL, and nerve growth factor receptor were observed in melanoma cell lines [20,21]. Studies on colorectal cancer revealed a small subset of preexisting quiescent LGR5 (leucine-rich repeat-containing G-protein-coupled receptor)-positive stem-like tumor cells, which were enriched after chemotherapy and were the source of relapse [22]. On the other hand, the results of many experiments in multiple tumor types and mathematical modeling indicate that the persister phenotype primarily emerges via plasticity induced by drug treatment [20,23,24,25,26].

The emerging concept of drug-tolerant persister (DTP) cells, a transient, non-genetically drug-resistant subpopulation of cancer cells capable of surviving initial therapy and driving disease relapse, was discussed in detail in previously published reviews [17,18,27,28]. The authors of these excellent studies focused on the characteristics of drug-tolerant persister cells, which is challenging due to the newly arising information. Additionally, these reports discussed the survival mechanisms of DTPs and sought to identify their vulnerabilities, which could be key points for therapeutic strategies. This field of science is emerging, and these reviews collect information from recent years (dated 2023–2025), making them valuable articles to study [17,18,27,28].

Recent developments demonstrate many examples of how nanotechnology can be used in the diagnosis and treatment of tumors. In the most recent reviews, the advantages, progress, and challenges of nanomedicines from preclinical studies to clinical markets were described [29,30,31]. In this review, we focused on the idea of whether nanomaterials can help combat DTPs. However, to define the potential nanotechnology application, we briefly summarize the specific characteristics and mechanisms of the DTPs that lead to their survival. Subsequently, we highlight recent advances and emerging strategies developed to target and eliminate this cell subpopulation, thereby enhancing the overall efficacy of cancer therapeutics. Thanks to the analysis of present anti-DTP strategies, we identified recent examples of using nanotechnology to target vulnerabilities typical of DTPs. Nanomaterials, with their unique physicochemical properties, offer a promising avenue for eliminating cancer cells, including DTPs, by enabling targeted drug delivery, controlled drug release, and modulation of cancer cell survival pathways. Collectively, these strategies may prevent tumor recurrence and significantly improve long-term patient outcomes.

## 2. Key Characteristics of Drug-Tolerant Persister (DTP) Cells

DTP cells can reprogram various cellular functions and alter the surrounding microenvironment to enhance their survival. This adaptability in both phenotype and genotype is considered a significant factor in tumor recurrence during cancer treatment [16,32]. The regulatory mechanisms involved in the formation of DTPs include cell reprogramming, phenotypic adaptation, modulation of the tumor microenvironment, and genetic adaptation.

**Figure 1:**
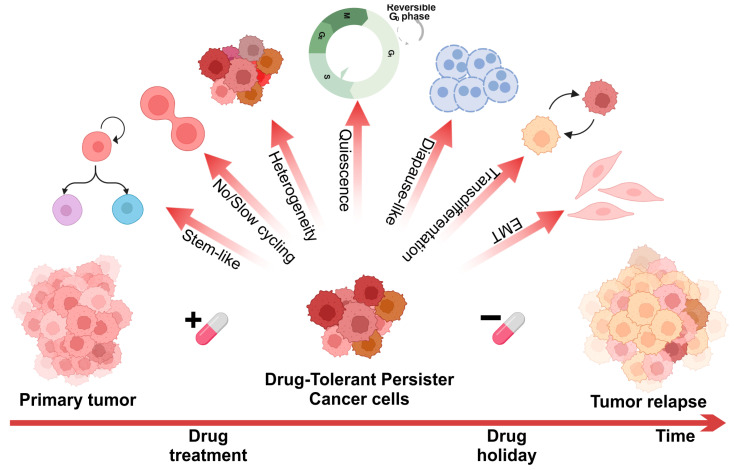
Hallmarks of drug-tolerant persister cancer cells: the most common features that sustain DTP survival during anti-cancer therapy and pose a risk of tumor recurrence.

Although the origin, phenotypic characteristics, and identification markers of DTPs are still under investigation, there are some common features associated with persister cells (Figure 1). These include a non-genetically driven phenotype, quiescence or proliferation arrest, and reversible drug sensitivity. Depending on the specific types of cancer and treatment strategies employed, DTP cells may exhibit additional features, such as incomplete apoptosis, ferroptosis, pyroptosis, genomic instability, a diapause-like status, and phenotype switching [18]. Furthermore, DTP cells that emerge during interaction with a specific drug do not form a uniform population. Instead, individual cells may employ different transcriptional programs, possess varying proliferation capacities, or exist in distinct metabolic states.

While most cancer persisters remain arrested in the presence of the drug, a rare subset can re-enter the cell cycle under constitutive drug treatment. Oren et al. found that cycling and non-cycling persisters come from different cell lineages with distinct transcriptional and metabolic programs [33]. Cycling persisters showed upregulation of antioxidant genes and a shift to fatty acid oxidation in NSCLC, breast cancer, and melanoma. Non-cycling cells, however, were characterized by increased Notch signaling and type I interferon-related genes. Quiescence is a notable characteristic of DTPs, but this state is temporary and occurs after drug treatment, unlike tumor dormant cells or senescent cells, which are often present in primary tumors and do not depend on treatment-induced stress. The reversible resting state of DTP is similar to stem cells. Cancer stem cells (CSCs) are defined by self-renewal, pluripotency, and maintenance in a quiescent slow-cycling state [11,34]. CSCs are pivotal in the persistence and recurrence of tumors due to their ability to self-renew and differentiate into various cell types found in a particular cancer. They are inherently resistant to traditional therapies, contributing to the survival of cancer cells post-treatment. They express specific markers, including CD133, CD44, CD166, CD24, and ALDH. Similarly, expression of these stem cell markers has been reported in DTPs derived from various types of cancers, such as ALDH, CD133, and CD24 in NSCLS [11,35,36], SOX2, OLIG, and NFIA in glioblastoma [37,38], and JARID1B and CD271 in melanoma [14,39]. Stem cell markers were also detected in breast cancer, gastric cancer, and bladder cancer [19,40,41]. However, in other types of cancer, including AML (Acute Myeloid Leukemia) and TNBC [42], no markers characteristic of CSCs were identified within the DTP population. Therefore, the phenotype is not a consistent feature of DTPs but rather indicates the ability of DTPs to exhibit cellular plasticity when subjected to drug-induced stress. DTPs exhibit remarkable plasticity, which allows them to survive under conditions of drug-induced stress. This adaptability is manifested through processes like epithelial-to-mesenchymal transition (EMT), transdifferentiation, and diapause-like states [27].

EMT is a biological process where epithelial cells shed their cell–cell adhesion and polarity to gain migratory and invasive properties, transitioning to a mesenchymal phenotype. This process involves multiple biochemical changes, resulting in the cells adopting fibroblast-like morphology and increased migration capacity, which is triggered by signals from the surrounding microenvironment. The process of EMT is intricately linked to drug resistance in cancer cells, contributing to cancer progression and invasiveness [43]. Markers such as vimentin, ZEB, TWIST, and SLUG were seen in EGFR-mutated lung cancer cells upon EGFR TKI (Tyrosine Kinase Inhibitor) therapy, underscoring the role of phenotypic plasticity in therapeutic resistance [44,45,46]. Activation of the EMT program has also been documented in DTP models of lung, breast, and ovarian cancer [13,47,48,49].

Transdifferentiation is a survival strategy employed by DTPs, involving the reprogramming of cancer cells into different lineages. This enhances their adaptability, allowing them to endure and adapt to therapeutic pressures. Drug-induced transdifferentiation has been documented in metastatic colorectal cancer cells that survive EGFR inhibition. These persister cells developed a Paneth cell-like phenotype, with the reprogramming process being mediated by the inactivation of Yes-associated protein (YAP), which serves as a master regulator of intestinal epithelium recovery from injury [50]. After treatment with androgen receptor inhibitors, prostate cancer cells can switch to AR-independent growth and survival pathways. This process, driven by epigenetic changes or signals from the tumor microenvironment, leads to neuroendocrine transdifferentiation [51]. These aggressive tumors, known as neuroendocrine prostate cancer (NEPC), reactivate developmental programs linked to epithelial–mesenchymal plasticity and stem-like properties. In a mouse model of basal cell carcinoma (BCC), residual BCC cells under vismodegib treatment exhibited a transcriptional program similar to stem cells of the interfollicular epidermis and isthmus, while untreated BCCs resembled hair follicle bulge cells [52].

Diapause-like status, as observed in colorectal cancer models, is another feature of DTPs [53]. Embryonic diapause is observed in over 130 species of mammals, where there is a reversible pause in embryo development, allowing birth to occur at a more advantageous time for survival [54]. During diapause, every cell in the embryo uniformly enters a reversible state and, upon resolution of environmental stress, resumes normal development. Rehman et al., using barcode analysis and mathematical modeling, demonstrated that colorectal tumors that entered the DTP state and recurred after treatment cessation did not lose clonal complexity [53]. Moreover, these data suggested that all cancer cells, rather than a small subpopulation, have an equal capacity to become DTPs. The initiation of embryonic diapause transcriptional programs, characterized by the downregulation of *MYC*, has also been observed in breast cancer, prostate cancer, and AML cells in the DTP state as a survival mechanism against chemotherapy or targeted therapy [55,56,57]. This reversible developmental arrest allows cancer cells to halt their growth and division in response to unfavorable conditions, resuming normal activity once the stressor is removed.

The combination of these processes—EMT, transdifferentiation, and diapause-like states—highlights the incredible adaptive capabilities of DTPs, contributing to their resilience and persistence in the face of anti-cancer therapies.

## 3. Therapeutic Strategies Targeting DTP Survival Mechanism

**Figure 2:**
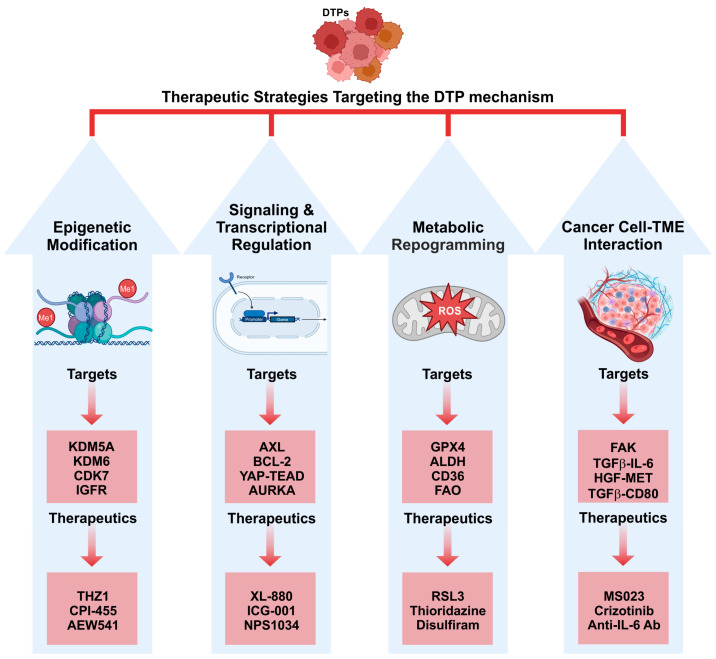
Therapeutic interventions targeting the survival mechanisms of DTP cancer cells, including epigenetic modification, signaling/transcriptional regulation, metabolic reprograming, and cancer cell–TME interaction.

To target DTP cells, it is essential to identify and target their induced survival mechanism. DTP cells survive under therapeutic pressure through various processes, including reversible phenotypic reprogramming, cell-cycle arrest, stemness, altered metabolism, and microenvironmental adaptation. These non-genetic mechanisms create a highly interconnected drug-tolerance network maintained by epigenetic modifications, signaling/transcriptional regulation, metabolic reprogramming, and cancer cell-TME interactions. Targeting key molecules regulating the mechanisms of drug tolerance can be an effective strategy in the treatment of DTPs and cancer recurrence (Figure 2).

### 3.1. Targeting DTP Epigenetic Modifiers

Epigenetic mechanisms like histone modification, DNA (Deoxyribonucleic Acid) methylation, and non-coding RNAs, by regulating patterns of gene expression, are essential to normal cellular development and differentiation. There is increasing data emphasizing the significance of epigenetic modifications in cancer drug-tolerant persister cells, as an important mechanism to evade therapeutic interventions [11,37,58,59]. As discovered by Sharma et al., EGFR-mutant NLCLC drug-tolerant persister cells required the histone demethylase KDM5A to establish a metastable chromatin state and maintain reversible drug tolerance and viability [11]. While KMD5A knockout significantly reduced the number of DTPs and DTEPs upon EGFR TKI treatment, transient expression of KDM5A decreased cancer cell sensitivity to treatment. As reported later, pretreatment of cancer cells with KMD5-specific inhibitor CPI-455 followed by targeted agents or standard chemotherapy led to the eradication of DTPs in multiple cancer cell line models, including NSCLC, melanoma, and breast cancers [12]. In addition to histone demethylases like KMD5A, histone deacetylases (HDACs) play an important role in the survival of DTPs. It has been shown that acetylation of histone H3K14 was significantly reduced in drug-tolerant cells, with a parallel increase in KMD5A expression and altered HDAC activity [11]. Studies have shown that HDAC activity is required for the maintenance of H3K9me3-mediated heterochromatin [60], which characterizes the repressed chromatin state of DTPs. It was observed that an increase in H3K9me3 in DTPs was most prominent over long interspersed repeat element 1 (LINE-1) [61]. HDAC inhibitors disrupted the H3K9me3-mediated heterochromatin state over LINE-1s, resulting in induction of their expression in the DTP population, leading eventually to the eradication of DTPs.

### 3.2. Targeting DTP Cell Signaling and Transcriprional Regulation

DTPs exhibit up-regulation of the kinase receptor genes (e.g., IGF-1R, AXL, FGFR3) and transcription-activating pathways (e.g., Wnt/b-catenin, YAP-TEAD) [62]. These transcriptional activations are involved in developing the DTP phenotype and sustaining DTPs. Therefore, inhibiting selected signaling pathways can be an effective treatment method to combat DTPs. In anaplastic lymphoma kinase–positive non-small cell lung cancer (ALK + NSCLC), treatment with ALK-TKI alectinib results in DTP formation [63]. Dual targeting of the ErbB pathway with the pan-HER inhibitor and Wnt/β-catenin pathway with tankyrase1/2 inhibitors (TNKS1/2) almost completely prevented the appearance of alectinib-induced DTP cells in ALK + NSCLC [63]. Kawakami et al. observed that gastric cancer treatment with 5FU leads to enrichment of residual cells overexpressing aldehyde dehydrogenase 1A3 (ALDH1A3), which was identified as a key survival factor of drug-tolerant persister cells in gastric cancer [64]. One of the downstream mediators of ALDH1A3 is the mTOR pathway, which is involved in cancer cell survival and proliferation. Inhibition of the ALDH1A3-mTOR axis with an mTOR inhibitor, temsirolimus, eradicated 5FU-tolerant gastric cancer cells [64].

### 3.3. Targeting DTP Metabolic Modifiers

The unique vulnerability of drug-tolerant persister cancer cells is highly dependent on mitochondrial respiration and the antioxidant enzyme GPX4 (Glutathione Peroxidase 4) for survival [13,65]. Data from cell line models and immunodeficient mice revealed that targeting and inhibiting of GPX4 resulted in ferroptotic death of DTPcells. Hence, blocking GPX4 can be a promising strategy towards the eradication of persister cells and the recurrence of the disease. The first and one of the most studied inducers of ferroptosis is erastin; its ability to trigger ferroptosis has been shown in various cancer cells lines [66], but its limited solubility remains a significant constraint in clinical application. The iron-dependent mechanisms underlying the persister cancer cell state, the main inducers of iron-dependent cell death, and the potential therapeutic applications are described in detail by Rodrigez et al. [65].

Dysregulation of metabolism in cancer persister cells increases the production of ROS (reactive oxygen species), leading to the dependence of these cells on efficient antioxidant pathways to reduce oxidative stress and ROS toxicity [65,67,68]. Raha et al. reported that increased expression of ALDH protected cells against the toxic effects of high levels of ROS in various models of DTP, including breast, lung, colon, and gastric cancers, after targeted treatment [19]. Inhibition of ALDH with disulfiram resulted in the accumulation of ROS to toxic levels, subsequent DNA damage, and apoptosis of drug-tolerant cells. Combining ALDH inhibition with targeted cancer therapeutics delayed treatment relapse in vitro and in vivo, revealing a novel combination treatment strategy.

### 3.4. Targeting DTP Cell–TME Interaction

The TME influences tumor growth, spreading, and response to anticancer treatment. Tumor cells, including DTPs, cross-talk with the TME components like cancer-associated fibroblasts (CAFs), tumor-associated macrophages (TAMs), endothelial cells, mesenchymal stem cells, immune cells, and the extracellular matrix via paracrine loops, chemokine networks, and direct interactions [28,62]. Cancer cells are subjected to challenging conditions within the tumor microenvironment (TME), including hypoxia, acidity, and nutrient deprivation, which drive metabolic reprogramming and epigenetic modifications in cancer cells, and together with drug pressure, result in the selection of metabolically reprogrammed DTPs [69]. Therapeutic strategies disrupting DTPs and TME interactions can significantly influence their survival. Straussmann et al. reported that in BRAF-mutant melanoma under RAF inhibition, the stromal secretion of hepatocyte growth factor (HGF) activates MET (Mesenchymal—Epithelial Transition factor) signaling, reactivation of the MAPK (Mitogen Activated Protein Kinase) and PI3K/AKT pathways, and immediate resistance to RAF inhibition [70]. Blocking the HGF-MET axis with crizotinib reversed cellular drug resistance and restored sensitivity to BRAF inhibition. Similarly, Sun et al. observed that HGF and FGF2 (fibroblast growth factor) significantly promoted the emergence of persisters, further indicating that they are important targets for anti-DTP therapy [71]. Moreover, they found that interferon-γ (IFNγ), which is released in the tumor microenvironment primarily by various immune cells, can induce a pro-persistence signal that is dependent on the signal transducer and activator of transcription 1 (STAT1), and IFNγ/STAT1-mediated persistence can be specifically eliminated by inhibition of type I protein arginine methyltransferase (PRMT) with MS023. Specifically, PRMT inhibition enhances the activity of P-STAT1 while decreasing the protein levels of STAT1, which is beneficial because P-STAT1 inhibits IFNγ-modulated persistence, while reducing STAT1 protein levels eliminates the pro-persistence effects of IFNγ [71].

Characteristics and molecular mechanisms of cancer DTP cells, as well as their vulnerability and therapeutic strategies, have been described in depth in recent reviews [17,18,27,28]. Additionally, the examples of therapeutic strategies to eliminate DTPs in cancer treatment are summarized in Table 1.

## 4. Application of Nanotechnology for Cancer Treatment

The application of chemotherapy to treat cancer is associated with a wide range of side effects. The primary issue is the cytotoxic impact of the drug on healthy cells. To minimize these obstacles and develop more effective treatments, intensive research is being conducted to generate novel formulations using various nanomaterials [75,76,77]. The unique structural and chemical properties of nanomaterials offer benefits such as improved drug bioavailability, slower drug release, extended circulation time, and reduced cytotoxicity to healthy cells [78,79]. The high surface-to-volume ratio of nanoparticles (NPs) makes them useful for delivering multiple therapeutic agents to cells [80]. Moreover, regulating the surface charge of nanoparticles plays a crucial role in controlling their cellular uptake, biodistribution, and membrane interaction, thereby influencing tumor accumulation, penetration and, ultimately, the overall therapeutic efficacy [81,82]. Additionally, the optical properties of some nanoparticles allow for tracking the biodistribution and accumulation of the drug, with the ability to visualize and quantify drug release [83,84]. As a result, nanoparticles have the potential to improve drug therapeutic efficacy while minimizing its side effects. Moreover, nanoparticles can benefit from enhanced permeability and retention effect (EPR); due to the leaky vasculature and impaired lymphatic drainage in the tumor microenvironment (TME), the passive accumulation of nanoparticles within tumor tissues can be obtained [85,86].

Various nanostructures, such as liposomes, dendrimers, nano-diamonds, quantum dots, peptides, cyclodextrins, carbon nanotubes (CNTs), graphene, and metal-based nanoparticles, are employed for diagnostic and therapeutic purposes [87]. For instance, gold nanoparticles are being tested in various formulations due to their advantageous properties, including biocompatibility and improved bioavailability and biodistribution [88,89]. Additionally, they can serve as a marker to monitor drug distribution within tissue and cells. Gold NPs also possess the typical property of localized surface plasmon resonance (LSPR) at the near-infrared (NIR) wavelength band, inducing less damage and demonstrating better tissue penetrability [90]. Iorn oxide nanoparticles (IONPs) are highly useful for cancer therapy due to their imaging capabilities with MRI and their magnetic properties, which are explored in targeted therapy and hyperthermia treatment. Additionally, IONPs can release iron ions, which can induce ferroptosis and/or promote the generation of reactive oxygen species (ROS). IONPs can also exert an immunoregulatory effect by stimulating the immune system to target cancer cells more effectively [91]. Liposomes, the first colloidal drug carriers approved by the FDA (Food and Drug Administration) [92], have been widely used for the delivery of both natural and synthetic chemotherapeutics [93,94]. Block copolymers, facilitated with tunable architecture, excellent stability, biocompatibility, and stimuli-responsive release capabilities, serve as highly effective platforms for advanced drug delivery and therapeutic applications [95,96].CNTs, along with other members of the fullerene family and carbon allotropes, are being explored as drug delivery systems [97], which facilitates efficient intracellular uptake, potentially leading to cell death [98,99]. However, the water insolubility of CNTs remains a significant limitation for their biological applications [100]. Numerous studies have shown the use of different nanomaterials in combination with various chemotherapeutic agents. Such a strategy has great potential to combat cancer, and it was described in detail in several recent review papers [101,102,103]. We would like to emphasize that the application of nanotechnology may be particularly important in the case of difficult-to-treat cancer cells, including drug-tolerant/resistant cells.

### 4.1. Application of Nanotechnology to Combat Persister Cancer Cells

As mentioned above, drug tolerance may manifest itself through regulatory mechanisms that have no genetic basis. The drugs that target epigenetic modifications, signaling/transcriptional regulation, metabolic remodeling, and DTP-TME interactions in drug-tolerant cells can be administered by nanoparticles. Due to nanoparticle attributes, the controlled delivery and activity of the drug can be achieved. Of special importance is targeted drug delivery. As reviewed in the previous section, drug-tolerant persister cells exhibit stem-like properties, including the overexpression of specific surface markers such as CD133, CD44, CD166, CD24, and CD271. By functionalizing the surface of nanoparticles with aptamers, peptides, antibodies, or small molecules that exhibit a strong affinity for specific surface markers [104,105], the targeted delivery of drugs embedded in NPs to DTP is potentially possible. Since the benefits of cancer monotherapy are rather limited, the application of nanotechnology can also enable the precise delivery of two or more drugs to achieve a synergistic effect, which is of special importance to combat the drug-tolerant cells. Most studies describe NPs that deliver two molecules (or more); one that kills directly the cancer cell and another that enhances the therapeutic outcome by targeting, e.g., cellular metabolism, signaling pathways, or efflux pumps. This approach can be critical in combating persistent cancer cells. Treating a tumor with a chemotherapy agent that is effective against most cancer cells would be ineffective if the cell developed a DTP-like phenotype. However, simultaneous delivery of a chemotherapeutic agent and a molecule that targets the mechanism responsible for developing drug tolerance could sensitize DTPs to the chemotherapy. Combining the specific targeting of NPs to DTPs, as mentioned above, may contribute to enhancing the effectiveness of anti-DTP drug delivery and, consequently, therapy. Moreover, while nanoparticles can be tested for their potential as drug delivery systems (DDS), other properties of the particles may also prove useful in combating persistent cancer cells, for example, through their direct effect on the cellular metabolism. For this type of approach, we can pay particular attention to NPs that possess properties that induce ferroptosis or activate ROS. Among the NPs with such characteristics, we can mention iron oxide- or graphene-based nanoparticles. These NPs not only can exert their properties within the tumor microenvironment but, once loaded with a drug, also can act as drug carriers. Additionally, we can decorate these NPs with various molecules to target DTPs specifically.

In the literature, we found no examples of articles addressing nanoparticle-based delivery of anti-persistence agents in the treatment of DTP cancer cells. Understanding the nature of cancer cell persistence and developing effective strategies to combat this problem is a relatively new field of research, and solutions will likely emerge in the near future. However, scientists have already tested several molecules considered anti-persistence agents for cancer treatment, delivered either within NPs or with NPs, but in the context of general cancer treatment (without specifically targeting drug-tolerant persister cancer cells). We summarize some examples of such studies below and in Table 2. Molecules considered as drugs against DTP cancer cells can be delivered by NPs, demonstrating that in the future, it will be possible to directly investigate the nanoparticle-based delivery of these drugs in the context of targeting DTPs.

#### 4.1.1. Nanotechnology in Targeting DTP Epigenetic Modifiers

As described earlier, the persistence of the cancer cells may be the result of epigenetic changes. Using nanoparticles to deliver drugs that target epigenetic mechanisms may be one approach to reversing the persistence state of cancer cells. Active targeting has become a new approach to targeting tumor cells precisely. In this strategy, the prime mechanism of action is to target the receptors that are overexpressed in the tumor cells. In the ovarian epithelium, follicle-stimulating hormone receptor (FSHR) is often overexpressed [106]. This overexpression further drives the crucial cancer cell characteristics, such as increased proliferation, metastasis, and angiogenesis in tumor cells [107]. Bhardwaj et al. applied the synthetic FSH β (33–53) peptide to deliver drugs to ovarian cancer cells overexpressing FSHR specifically [108]. The FSH β (33–53) peptide was linked to PEGylated graphene oxide (pGO) nanoparticles that were loaded with two drugs: Gambogic acid (GA) to induce cellular apoptosis and Jun Qi 1 (JQ1) for epigenetic modification. The JQ1 molecule is an epigenetic inhibitor that targets the bromodomain proteins, preventing acetylation of histone residues on the chromatin. Its activity was previously investigated in DTP cancer cell treatment cancer cells treatment [49]. The developed system was named pGO-FSH-JQ1-GA and used to treat ovarian cancer cells Caov-3, significantly enhancing cellular apoptosis. Further, transcriptome sequencing studies highlighted that SMAD2 (mothers against decapentaplegic homolog 2) was significantly downregulated by JQ1 and GA [109]. Overall, the developed system was found to be a promising active targeting strategy to deliver drugs to the FSHR-overexpressing cells and showed that the JQ1 molecule can be incorporated into NPs, retaining its activity.

Masoudi et al. developed a novel nano-carrier system, Ch-J-NPs, comprised of a biocompatible polymer chitosan and an inhibitor JQ1 for epigenetic regulation [109]. It was indicated that JQ1 delivered in the chitosan-based nanoparticles affected the cell cycle and apoptosis in OVCAR3 ovarian cancer cells. The authors studied the expression levels of genes involved in the cell cycle and apoptosis, indicating a reduction in genes related to cell cycle progression and an increase in genes responsible for apoptosis [109]. Hence, the study also reported the successful delivery of an active JQ1 inhibitor by NPs; Ch-JQ1-NPs can also be used to treat DTP cancer cells.

Another approach in combating cancer is to use the specific properties of nanoparticles in combination with molecules that regulate epigenetics. Nóra Igaz et al. demonstrated the combinational effect of Histone deacetylase (HDAC) inhibitors and silver nanoparticles (AgNPs) against cervical cancer HeLa cells [107]. This combination works synergistically; the HDAC inhibitor, Trichostatin A (TSA), produces a hyperacetylation of the chromatin and subsequently reduces the interaction between histone proteins and the genomic DNA, causing chromatin relaxation. Since the therapeutic effect of AgNPs relies on generating reactive radicals (ROS), DNA damage, and apoptosis in cancer cells, the relaxed chromatin structure can be more prone to damage caused by AgNPs. The researchers studied molecular events with single compounds and combination administrations to understand this synergistic mechanism. AgNPs did not influence the HDAC-inhibiting capability of TSA. Moreover, after the co-administration of AgNPs and TSA, a significant quantity of ROS was generated that impacted the cellular events; TSA facilitated the DNA-damaging and apoptotic potential of AgNPs. The combinational effect of TSA and AgNPs led to enhancement in the formation of double-strand DNA breaks. Hence, the study concluded that the combinational treatment was more effective in combating cervical cancer than single compounds [107]. These results indicated that nanomaterials can be used in combination with molecules that control epigenetic modifications in novel therapeutic applications. The drug-tolerant persister cancer cells can be potentially targeted in such therapy, primarily because two important mechanisms leading to acquiring persistence can be addressed: metabolic reprogramming and epigenetic modifications.

A similar approach was studied by Xi-Feng Zhang et al.; the combinational effect of reduced graphene oxide-silver nanoparticles (rGO-AgNPs) and TSA to treat human ovarian cancer cells (SKOV3) was examined [110]. A novel material, lycopene, was used in the development of nanocomposites. Graphene oxide (GO) was used to stabilize AgNPs, preventing aggregation and facilitating the controlled release of Ag+ ions. The various assays, including cell viability, cytotoxicity, mitochondrial membrane permeability, DNA fragmentation, and DNA double-strand breaks (DSBs), evidenced that the combination of low doses of rGO-Ag and TSA caused high cytotoxicity and induced greater apoptosis in SKOV3 cells than single agents administered at high concentrations [110]. The treatment outcomes resulted from various mechanisms, including reactive oxygen species generation, mitochondrial dysfunction, and DNA damage. Such approaches can potentially be of special importance in overcoming tumor persistence.

Co-administration of siRNA silencing EphA2 (siEphA2) loaded into cationic solid lipid nanoparticles (cSLNs) and the histone lysine demethylase inhibitor (JIB-04) was studied in a prostate cancer model [111]. EphA2 is a member of the receptor tyrosine kinase (RTK) family and is generally overexpressed in various cancer types, including breast, lung, prostate, and brain. Moreover, the overexpression of the histone lysine demethylase family members is also observed in many tumors and has been shown to be responsible for drug resistance by promoting epigenetic modifications. In the study by Oner et al., first, the researchers developed a cSLN containing dimethyl di-octadecyl ammonium bromide (DDAB) loaded with siEphA2 (DDAB-cSLN/siEphA2), which achieved a higher cellular uptake and gene silencing effect than the other NPs, DOTMA(1,2-di-O-octad ecenyl-3-trimethylammonium-propane)-based SLPs (Synthetic Lipoproteins). Although DDAB-cSLN/siEphA2 showed a remarkable EphA2 silencing effect at both mRNA and protein levels in prostate cancer cells PC-3, the anti-cancer effect was relatively limited; silencing EphA2 did not cause any significant change in the cell viability, migration, and colony formation abilities of PC-3 cells. In the next step, the co-administration of DDAB-cSLN/siEphA2 with JIB-04 was studied, indicating the significant effect of JIB-04 on the expression level of *EphA2* and the silencing effect of DDAB-cSLN/siEphA2. In general, the JIB-04 inhibitor decreased *EphA2* expression and increased the silencing efficiency of DDAB-cSLN/siEphA2. For the first time, the role in gene expression regulation of JIB-04 was highlighted [111]. This group provided an efficacious delivery system that can be studied further to target other genes and cancer types. Although the JIB-04 inhibitor was examined as a co-administered molecule without any carrier, the studies are a good example of combinational therapy, combining nanoparticle-based drug delivery with molecules that regulate epigenetic modifications.

#### 4.1.2. Nanotechnology in Targeting DTP Cell Signaling and Transcriptional Regulation

One potential therapeutic approach to combating DTPs can be reprogramming the cell signaling network and regulating gene transcriptomics. Small-molecule inhibitors are drugs commonly used in cancer treatment that interfere with specific cell signaling pathways inside cancer cells. They inhibit specific proteins, such as kinases, to modify/kill cancer cells or to overcome cancer cell resistance. The application of NPs can improve their efficiency. T. Zhong et al. formulated the dual drug delivery system of sorafenib (SORA) and crizotinib (CRIZ) using biodegradable triblock poly(ethylene glycol)–poly(e-caprolactone)–poly(ethylene glycol) (PEG–PCL–PEG, PECE) [112]. SORA and CRIZ are protein kinase inhibitors widely used in cancer treatment; however, their low solubility can be problematic. The hydrophilic (PEG) and hydrophobic (PCL) blocks of PECE self-assembled in controlled conditions, entrapping SORA and CRIZ and generating SORA–CRIZ@NPs. The obtained NPs displayed a small particle size, spherical shape, and narrow particle size distribution, and their internalization into 4T1 and A549 cancer cells was enhanced compared with SORA + CRIZ powder. Moreover, the in vivo study reported that SORA–CRIZ@NPs showed superior cytotoxicity, suppressing the tumor development and producing minor damage to essential organs compared with controls [112]. This study is an example of the application of NPs to deliver agents that can also be used against DTPs.

Korucu Aktas P et al. developed a novel crizotinib-loaded lipid-polymer hybrid nanoparticle (CL-LPHNPs) [113]. Two formulations were developed using different polymers (PLGA and PLC), which entrapped crizotinib with high efficiency; 79.25 ± 0.07% and 70.93 ± 1.81%, for CL-PLGA-LPHNPs and CL-PCL-LPHNPs, respectively. The fabricated particles effectively delivered the drug to non-small cell lung cancer cells (NCI-H2228), significantly reducing the cells’ viability compared with free crizotinib. Additionally, in NCI-H2228 cells, the gene expression and protein level of RAS, RAF, MEK, and ERK were lower after treatment with CL-PLGA-LPHNPs/CL-PCL-LPHNPs than after free crizotinib administration [113]. New formulations, optimized using the Box–Behnken design (BBD), have great potential for wider applications, including treating DTPs.

The studies mentioned above examined the direct effect of crizotinib administration on cell signaling pathways in cancer cells. However, this tyrosine kinase inhibitor has also been shown to effectively block the hepatocyte growth factor (HGF) and its receptor (MEF) pathway in cancer-associated fibroblasts (CAFs), which can be essential for modifying TME interactions (described in 3.4) [70]. Tyrosine kinase inhibitors can affect various cells in the TME, and nanoparticles may be helpful for targeted delivery of these highly reactive but relatively cell-type-insensitive molecules. The nanocomposite, THZ1@8P4 NPs, was developed to enhance the therapeutic efficiency of THZ1, a small molecule CDK7 inhibitor, in a gallbladder cancer (GBC) model [114]. THZ1 is responsible for downregulating CDK7-mediated phosphorylation of RNA polymerase II (RNAPII), which results in a significant downregulation of transcriptional programs and can also be important in DTP cells. However, the THZ1 molecule shows limitations, such as strong hydrophobicity, low bioavailability, and rapid metabolism in vivo. To overcome the mentioned shortcomings and improve the drug efficacy, a nanocarrier made of phenylalanine-based poly(ester amide) polymer (8P4) was employed (8P4 NPs). The THZ1@8P4 NPs indicated higher drug cellular uptake and escape from lysosomes than the free drug. Additionally, the in vitro study of THZ1@8P4 NPs against GBC cells indicated a more significant inhibitory effect than free THZ1; THZ1@8P4 NPs decreased the cell viability, increased cell apoptosis, caused cell cycle blocking in the G2/M phase, decreased the number of formed colonies, and reduced the mRNA expression of *FOSL1* (Fos-like antigen 1) and *JUN* (*Jun* proto-oncogene, AP-1 transcription factor subunit). The in vivo study indicated that the THZ1@8P4 NPs exhibited improved pharmacokinetics and superior antitumor effects, with negligible systemic toxicity. The THZ1@8P4 NP administration in a patient-derived xenograft model (PDX) caused a reduction in *JUN* and *FOSL1* expression and significantly inhibited tumor growth compared with free drug application. Hence, the study concluded that THZ1@8P4 NPs can target CDK7-mediated transcriptional addiction in GBC [114], and by extension, in other cancer models, including hard-to-treat DTPs.

#### 4.1.3. Nanotechnology in Targeting DTP Metabolic Modifiers

As mentioned above, metabolic reprogramming is crucial for tumor development. It provides cancer cells with the necessary energy and redox balance to sustain their uncontrolled growth and proliferation. Specific metabolic alterations are observed in DTP and cancer cells, and they constitute a potential target for therapy. The application of NPs for delivery agents interfering with cancer metabolism has already been explored.

Xun Jin et al. demonstrated the use of an amphiphilic polymer methoxy poly(ethylene glycol)-poly(l-lactic acid) (MPEG-PLA) for effective drug delivery in a breast cancer model [115]. The combinational therapy was developed by simultaneously delivering two drugs, doxorubicin (Dox) and thioridazine (Thio). Thio, a phenothiazine mainly used as an antipsychotic drug, was found to be responsible for inhibiting cellular energy metabolism [116]. By suppressing the metabolism, thioridazine effectively disrupts the survival mechanisms of persister cells. Dox is one of the most widely applied anticancer drugs for various malignancies [117]. Although the drug loading efficiency of Thio and Dox was relatively low (4.71% and 1.98%, respectively), MPEG-PLA NPs co-loaded with Thio and Dox exerted a better therapeutic effect on tumor growth inhibition than free Dox, free Thio, or the combination of free Dox/Thio in 4T1 breast cancer xenograft mice [115].

A formulation of a ferroptosis-promoting drug delivery system was developed using folic acid (FA)-modified iron oxide nanoparticles loaded with miR-214–3p (named FeNP/miR) to target hepatocellular carcinoma (HCC) [118]. FA demonstrates high affinity for folate receptors and is non-cytotoxic; hence, the modification of NPs with FA increases their cellular uptake, especially in cancer cells overexpressing folate receptors [119]. Further, the miR-214–3p is a key element in regulating iron metabolism and promoting ferroptosis, and it works using two mechanisms. First, it downregulates Lipocalin 2 (LCN2), a protein that is involved in various biological processes, including regulation of iron homeostasis by decreasing intracellular iron levels and stimulating the expression of glutathione peroxidase 4 (GPX4) [120]. By the downregulation of *LCN2* expression, there is a reduction in iron efflux and an enhancement in the Fenton reaction in cells. The second role of miR-214–3p comprises lowering the expression of GPX4, which weakens cellular antioxidant defenses by increasing lipid peroxidation and further inducing ferroptosis. The GPX4 is a key regulator of ferroptosis [121]. It belongs to the glutathione peroxidase family, which catalyzes the reduction of hydrogen peroxide, organic hydroperoxides, and lipid hydroperoxides, thereby protecting cells against oxidative damage. If the level of GPX4 decreases significantly, the accumulation of unrepaired lipid peroxides, lipid peroxidation, and ferroptosis occurs [121]. The FeNP/miR particles were very effective in cancer treatment [118]. Due to FA modification, the obtained particles effectively targeted tumors in vivo. Furthermore, the synergy between each composite component enhanced ferroptosis in liver cancer cells in vitro and in vivo. The proposed strategy is promising not only in targeting HCC but also in combating DTP cancer cells due to the downregulation of GPX4 [118].

Plasma technology, especially cold atmospheric plasma (CAP), has emerged as a new strategy for cancer treatment [122]. CAP’s main components, i.e., reactive oxygen and nitrogen species (RONS), lead to lipid peroxidation and cell death However, the GPX4 enzyme can protect cells against oxidative damage, potentially reducing CAP’s effectiveness against cancer cells. To enhance CAP’s activity, Cao et al. proposed combining CAP with RAS-selective lethal 3 (RSL3), a synthetic GPX4 inhibitor [123]. Moreover, to improve its water solubility and in vivo stability, a hydrophobic RSL3 was loaded in poly(ethylene glycol)-block-poly(lactide-co-glycolide) (PLGA-PEG) nanoparticles (named RSL3@NP). Indeed, the RSL3@NP amplified the anti-tumor efficacy of CAP via enhanced GPX4 inhibition and increased cancer cell death in vivo and in vitro. Furthermore, the developed method for the simultaneous delivery of RSL3@NPs and CAP using hydrogel resulted in cancer immunogenic cell death (ICD). The injectable hydrogel-mediated delivery of RSL3@NPs and CAP induced the promotion of dendritic cell maturation, the polarization of the M2 to M1 phenotype of TAMs, and the enhancement of T cell infiltration to the tumor, which improved the anti-tumor effect [123]. The application of NPs for the delivery of GPX4 inhibitors can also be further explored in combating DTP cancer cells.

Additionally, combined therapy can be exploited; the intrinsic properties of NPs that impact cellular metabolism and the drug’s activity can act synergistically. The combined effects of platinum nanoparticles (PtNPs) and doxorubicin (DOX) were evaluated in osteosarcoma (OS) U2OS cells [124] The PtNP–DOX combination exhibited a synergistic cytotoxic effect through increased lactate dehydrogenase (LDH) release and elevated reactive oxygen species (ROS) generation. PtNPs play a crucial role in enhancing oxidative stress and promoting DNA damage in OS cells; therefore, their co-administration with DOX represents a promising and potent therapeutic strategy for osteosarcoma treatment [124]. Similarly, the intrinsic properties of PtNPs, which can modify the cellular metabolism, may be applied in combined therapy for DTPs.

Moreover, Genc et al. indicated that the intrinsic properties of Fe_3_O_4_ NPs can enhance cancer therapy using 5-fluorouracil (5-FU). The synergistic effects of Fe_3_O_4_ NPs and 5-fluorouracil (5-FU) on Caco-2 colon cancer cells were observed [125]. Treatment with a Fe_3_O_4_-NPs+ 5-FU combination resulted in higher membrane penetration and cellular uptake of 5-FU. The co-treatment induced cytotoxic, immunomodulatory, and oxidative stress responses at concentrations where 5-FU alone had minimal impact on these cellular mechanisms observed [125]. Again, the iron oxide nanoparticles can be of special importance in combating DTPs. Its intrinsic properties, leading to modification of the cellular metabolism (the induction of ferroptosis and/or the generation of reactive oxygen species) and immunoregulation can directly target DTPs’ vulnerabilities and thus could constitute a great therapeutic strategy.

#### 4.1.4. Nanotechnology in Targeting DTP Cancer Cell–TME Interactions

As mentioned, TME has a unique cellular and molecular composition, which may be crucial for developing and surviving DTP cancer cells. Interactions between cancer cells and other components of the TME, such as immune cells and fibroblasts, can induce the cells’ persistence. Thus, the therapeutic effect can be accomplished or improved by blocking, disrupting, and/or modifying cell–cell interactions within the TME. The various molecules modifying the TME have already been explored, including those delivered by NPs; they can also be used to eradicate persister cancer cells in future studies.

Chin et al. demonstrated the formation of cluster-structured nanoparticles (CNPs) consisting of Fe_3_O_4_ and iron chlorophyll (Chl/Fe) photosensitizers for bladder cancer (BC) treatment [126]. To achieve effective cellular internalization by targeting glycoproteins on BC cells, CNPs were conjugated with carboxyphenylboronic acid (CPBA). The administration of photoactive Fe_3_O_4_@Chl/Fe CNPs resulted in the destruction of cancer cells by photodynamic therapy (PDT) and chemodynamic therapy (CDT)-mediated ferroptosis. On one side, due to PDT, the activated photosensitizer produced reactive oxygen species (ROS), while on the other side, the iron-containing CNPs induced the CDT-mediated Fenton reaction to produce hydroxyl radicals (•OH). The cellular redox balance was changed, which caused the killing of cancer cells. The Fe_3_O_4_@Chl/Fe CNPs activity caused the downregulation of GPX4, which constitutes an essential target in persister cancer cells. Moreover, the additional outcome was observed after application in vivo of the Fe_3_O_4_@Chl/Fe CNPs in a model of orthotopic MB49-bearing mice. The CNP-based therapy not only caused the inhibition of tumor growth but also resulted in the reduction of immunosuppressive factors such as PD-L1, IDO-1, TGF-β, and M2-like macrophages and in the induction of CD8+ T cells, as indicated by IHC (Immunohistochemistry) analysis of tumor samples. The group demonstrated that the developed nanoclusters can directly kill cancer cells and, more importantly, remodel the TME [126].

ALK-positive NSCLC initially responds to treatment with ALK tyrosine kinase inhibitors (TKIs) but relapses over time. ALK-positive NSCLC tumors belong to a group of “cold” tumors, characterized by a low immune cell count. In such cases, the application of TKIs and immunotherapies often proves ineffective. One possible solution in such a situation would be to generate an immunogenic environment in the tumor, e.g., by introducing reprogrammed macrophages. Bauer et al. developed glutathione-responsive core cross-linked polymeric micelles (CCPMs) to deliver iron to macrophages specifically [127]. The iron oxide nanoparticles were embedded in polymeric micelles of reactive polysarcosine-block-poly(*S*-ethylsulfonyl-l-cysteine). Then, NPs were functionalized with dihydrolipoic acid to allow iron oxide cores to cross-link and form disulfide bonds with the surrounding poly(*S*-ethylsulfonyl-l-cysteine) block. Upon internalization of the formed (Superparamagnetic iron oxide nanoparticles) SPION-CCPMs into macrophages, the cleavage of the disulfide cross-links in the nanoparticle core occurred, leading to sustained intracellular iron release, which induced a strong pro-inflammatory activation [127]. The study by Horvat et al. examined the application of SPION-CCPMs in the therapy of ALK-positive NSCLC tumors [128]. The detailed in vitro study indicated that accumulated SPION-CCPM in macrophages stimulated phagocytosis and the secretion of factors that reduced the proliferation and viability of co-cultured cancer cells. In an Eml4-Alk lung cancer mouse model, the administration of SPION-CCPMs reprogrammed tumor-associated macrophages to secrete reactive nitrogen species and cytokines and induced recruitment of CD8+ T cells, which finally resulted in the reduction of tumor growth. Furthermore, in lung cancer-bearing mice, after first-line therapy with crizotinib (one of the TKIs), the SPION-CCPMs significantly halted the regrowth of relapsing tumors [128]. The SPION-CCPMs–dependent rewiring of the phenotypes of iron-containing immune cells reshaped the immunosuppressive TME; it rewired the communication of the immune cells, which can be vital for removing residual DTP cancer cells. The study by Horvat et al. indicated the application of NPs to modify the cell–TME interaction in cancer but also specifically examined cancer treatment with crizotinib. The crizotinib was also examined as an inhibitor of DTP–TME interaction in the BRAF-mutant melanoma model [70]. The correlation between hepatocyte growth factor (HGF) expression in stromal cells (such as cancer-associated fibroblasts, CAFs) and innate resistance (persistence) to RAF inhibitor treatment of melanoma cells was indicated. Dual inhibition of RAF (by Vemurafenib) in melanoma and the HGF-MET pathway (by crizotinib) in CAFs resulted in reversal of the drug’s initial resistance. Thus, SPION-CCPMs combined with crizotinib may serve as a dual therapeutic strategy by targeting both immune and stromal cells, particularly in persister cancer, though further investigation is required.

We presented and described several examples of NP application for delivering drugs that target epigenetic modifications, cell signaling/transcriptional regulation, metabolic remodeling, and DTP cell-TME interaction in cancer treatment. Most of the examples are recent, indicating the development of drug delivery systems that can be tailored to the special requirements of the cancer cells and TME. More examples are listed in Table 2. Although the selected articles do not directly address the treatment of drug-tolerant persister cancer cells, the described drugs have previously been studied as agents indicating the therapeutic vulnerabilities of cancer drug-tolerant persister cells [27]. The application of NPs for their delivery can potentially enhance their therapeutic outcome.

**Table 2:** Application of nanoparticles for the delivery of anti-DTP drugs in cancer therapy. Nanocarriers enhance drug accumulation in tumors, improve drug efficacy, and may increase the effectiveness of therapies targeting drug-tolerant cancer cells.

Nanomaterial/ Delivery System	Cancer Type/Model	Combination/ Co-Therapy	Key Findings/Mechanism	Ref.
Bovine serum albumin nanoparticles (BSA NPs)	Colorectal cancer (c-Met positive)	Crizotinib-IR808 conjugate	Enables NIR-II imaging-guided chemo-photo- therapy; targeted tumor delivery; synergistic chemo-phototherapy under laser irradiation	[129]
Mitochondria-targeting lipid-polymer hybrid nanoparticles PLGA/CPT/DSSP (poly(d,1-lactide-co-glycolide)/C18-PEG2000-TPP/DLPE-S-S-mPEG4000)	Breast cancer (MCF-7 cells)	RSL3 (GPX4 inhibitor) + Artemisinin	Synergistic apoptosis and ferroptosis; RSL3 enhances artemisinin-induced cell death via GPX4 inhibition	[130]
Mesoporous silica nanoparticles (MSN) with chitosan and lactobionic acid	Hepatocellular carcinoma (HCC)	Sorafenib + Ursolic acid	Enhanced tumor targeting, pH-responsive release, synergistic inhibition of growth and metastasis, increased apoptosis	[131]
Cancer cell–platelet hybrid membrane- camouflaged lipid nanoparticles	Hepatocellular carcinoma (HCC)	Sorafenib + Triptolide	Long circulation, tumor targeting, synergistic tumor inhibition, reduced sorafenib dose and toxicity	[132]
Ultra-small lipid nanoparticles (usLNPs)	Hepatocellular carcinoma (HCC)	Sorafenib + Midkine-siRNA	Overcomes sorafenib resistance, enhanced tumor accumulation, potent gene silencing, strong tumor eradication	[133]
Zwitterionic polymer-coated Fe_3_O_4_ nanoparticles	Colon cancer	Sorafenib + Fe_3_O_4_	Induces ferroptosis, extended circulation, enhanced tumor accumulation, strong tumor inhibition	[134]
Bismuth-based mesoporous nanomaterial (NBOF) coated with polyethylene glycol and folic acid conjugates(P-FA)	Hepatocellular carcinoma (HCC)	Sorafenib + NBOF	Synergistic photothermal and molecular therapy, enhanced imaging, significant tumor growth reduction	[135]
HDL-like organic-core lipid nanoparticles	Prostate, AML, pancreatic cancer	Multi-kinase inhibitor (PIK-750	SR-B1 targeted delivery, potent cell death, reduced tumor growth, and few side effects	[136]
Lipid nanoparticles (EGFR-PEG bispecific Ab)	Neuroblastoma	PLK1 siRNA (kinase silencing)	Enhanced targeting, gene silencing, and reduced tumor growth in vivo	[137]

## 5. Conclusions

**Figure 3:**
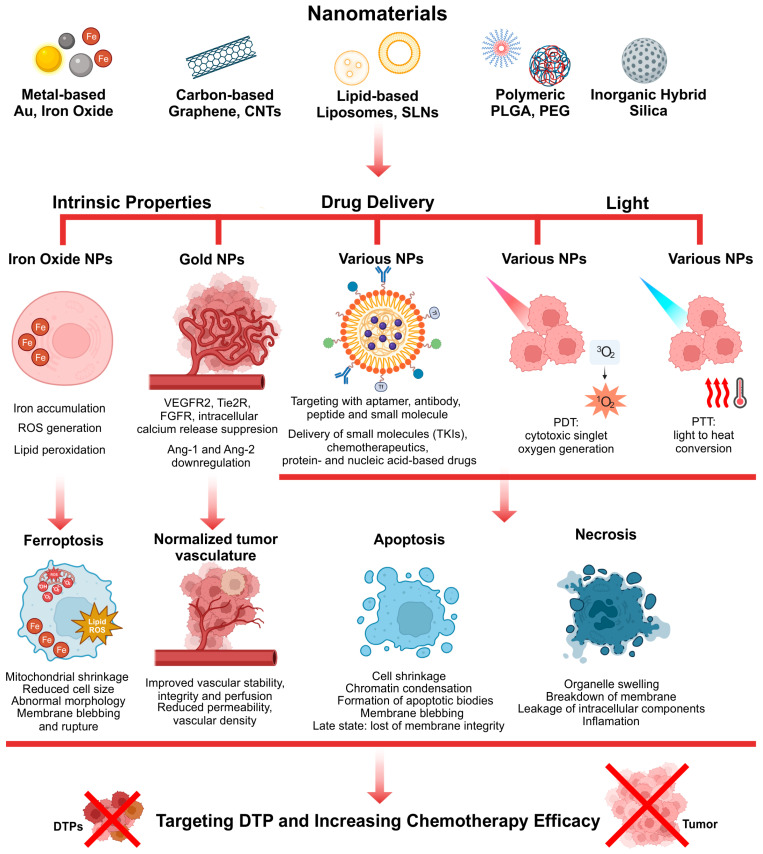
Nanomaterial platforms for potential therapy of drug-tolerant cancer cells, highlighting their intrinsic properties, potential for targeted drug delivery, and application to light-induced anticancer therapies.

Drug resistance in malignancies is increasingly attributed to the emergence of drug-tolerant persister cells. These cells survive under therapeutic pressure through different mechanisms, including reversible phenotypic reprogramming, cell-cycle arrest, stemness, altered metabolism, and microenvironmental adaptation. These non-genetic mechanisms create a highly interconnected resistance network maintained by cell–TME interactions, signaling pathways, transcriptional regulation, and redox homeostasis. A central challenge lies in dissecting and distinguishing the fundamental differences between “regular” cancer cells, transformed DTPs, and cancer stem cells. Elucidation of DTP biology through advanced technology coupled with robust preclinical models is essential for defining their heterogeneity and for distinguishing them from other resistant cell subpopulations such as cancer stem cells. Subsequently, eliminating DTPs may be achieved by targeting their key vulnerabilities, such as redox imbalance, ferroptosis sensitivity, and epigenetic modifications, as well as differences in cell signaling and transcription. To this end, integrative strategies that combine molecular targeting, adaptive therapy design, and modulation of the tumor microenvironment are essential. Within these combinational approaches, nanotechnology can play a pivotal role, with nanoparticles serving as versatile platforms for precise drug delivery and regulation of the microenvironment. Nanoparticle-based drug delivery systems (polymeric, metallic, hybrid, and multifunctional nanoplatforms) represent a particularly promising strategy to suppress drug-tolerant cancer cells by improving pharmacokinetics, enhancing intratumoral accumulation via passive and active targeting, and enabling co-delivery of chemotherapeutics, nucleic acid-based drugs, and immunomodulators (Figure 3). Moreover, immobilization of tumor-homing ligands, stimuli-responsive drug release, and integrated theranostic functionalities lead to enhancement of the anti-cancer therapeutic efficacy.

As highlighted in this review, the combination of nanomaterials with small molecules or nucleic acids demonstrates superior efficacy against tumor recurrence. However, their clinical translation remains constrained by immunotoxicity, long-term safety concerns, and the absence of predictive preclinical models. Moving forward, rational nanoplatform design, informed by a deeper understanding of surface functionalization, enhanced permeability and retention (EPR) dynamics, and tumor physiology, will be pivotal in establishing nanoparticles as clinically viable tools against drug-tolerant cancers. Figure 3 illustrates nanomaterial platforms that can be explored for the eradication of DTPs. The intrinsic properties of nanomaterials can induce cell ferroptosis and normalize tumor vasculature, light-induced NPs can generate/support DTP and DTT therapies, and NP-based drug delivery capacity can be explored in the targeted delivery of various types of drugs, including the targeting of DTP vulnerability. The combined application of DTP-oriented, NP-based therapy with chemotherapeutic molecules can overcome cancer cells’ persistence and prevent cancer relapse.

## Figures and Tables

**Table 1:** Therapeutic strategies to eradicate drug-tolerant persisters by targeting their survival pathways through epigenetic modulation, signal transduction/transcriptomic regulation, metabolic reprogramming, and cell-TME interaction to overcome persistence and prevent cancer relapse.

Cancer Types	Exp. Model	Treatment	Mechanism of DTP	Target of Therapy	Proposed Drug to Target DTPs	Ref.
NCLC	Cell lines; PC9, HCC4006	Osimertinib + trametinib	Transcriptional regulation	YAP-TEAD pathway	TEAD-inhibitor (MYF-01-37)	[44]
	Cell lines; PC9	EGFR TKI Erlotinib	Epigenetic modification and transcription regulation	H3K9 methylation over LINE-1 elements	HDAC inhibitor (MS275 or TSA)	[61]
	Cell lines; PC9, H1975, HCC827, HCC4006	EGFR TKI Erlotinib	Signaling pathway	FGFR3 signaling	Pan-FGFR inhibitor (infigratinib)	[45]
	Cell lines; PC9, HCC4011	EGFR TKI Osimertinib	Signaling pathway	AXL signaling	AXL inhibitor (NPS1034)	[72]
NCLC, GC	Cell lines; PC9, MKN45	EGFR TKI Erlotinib, crizotinib	Metabolic changes	ALDH	ALDH inhibitor (Disulfiram)	[19]
LUAD	Cell lines; A549, H460 and PDO	MEK inhibitor Trametinib	Metabolic changes	Mitophagy	Chloroquine	[73]
Melanoma	Cell lines; SK-Mel-5, SK-Mel-28, G-361	Vemurafenib	Tumor microenvironment	HGF/MET paracrine pathway	MET inhibitor crizotinib	[70]
BBC	PDX	Hedgehog inhibitor Vismodegib	Signaling pathway	Wnt signaling	Wnt signal inhibitor (LGK-974)	[74]

## Data Availability

No new data were created or analyzed in this study. Data sharing is not applicable to this article.

## Abbreviations

The following abbreviations are used in this manuscript:

•OH	Hydroxyl Radicals
Ab	Antibody
AEW541	Insulin-Like Growth Factor–Type 1 Receptor (IGF1-R) Inhibitor
AgNPs	Silver Nanoparticles
ALDH	Aldehyde Dehydrogenase
AML	Acute Myeloid Leukemia
Ang-1	Angiopoietin 1
Ang-2	Angiopoietin 2
AURKA	Aurora A Kinase
AXL	AXL Receptor Tyrosine Kinase
BBD	Box–Behnken design
BC	Bladder Cancer
BRAF	B-Raf Proto-Oncogene, Serine/threonine Kinase
Ca+2	Calcium
CAFs	Cancer-Associated Fibroblasts
CAP	Cold Atmospheric Plasma
CDT	Chemodynamic Therapy
CDK7	Cyclin-dependent Kinase 7
Chl/Fe	Iron Chlorophyll
CL-LPHNPs	Crizotinib-Loaded Lipid-Polymer Hybrid Nanoparticles
CNPs	Cluster-Structured Nanoparticles
CNTs	Carbon Nanotubes
CPBA	Carboxyphenylboronic Acid
CPI-455	Pan-KDM5 Inhibitor
CRIZ	Crizotinib
cSLNs	Cationic Solid Lipid Nanoparticles
DC	Dendritic Cell
DDAB	Dimethyl Di-octadecyl Ammonium Bromide
DDS	Drug Delivery System
DNA	Deoxyribonucleic Acid
DOTMA	1,2-di-O-octadecenyl-3-trimethylammonium-propane
Dox	Doxorubicin
DSBs	DNA Double-Strand Breaks
DTP	Drug-Tolerant Persister
EGFR	Epidermal Growth Factor Receptor
EPR	Enhanced Permeability and Retention effect
ERK	Extracellular Signal-regulated Kinase
FA	Folic Acid
FAK	Focal Adhesion Kinase
FAO	Fatty Acid Oxidation
FDA	Food and Drug Administration
FGF	Fibroblast Growth Factor
FGFR	Fibroblast Growth Factor Receptor
*FOSL1*	Fos-like Antigen 1
FSHR	Follicle-Stimulating Hormone Receptor
GA	Gambogic Acid
GBC	Gallbladder Cancer
GO	Graphene Oxide
GPX4	Glutathione Peroxidase 4
H3K9me3	Histone 3 Lysine 9 Trimethylation
HCC	Hepatocellular Carcinoma
HDAC	Histone Deacetylase
HGF	Hepatocyte Growth Factor
IC50	Half-maximal Inhibitory Concentration
ICD	Immunogenic Cell Death
IDO-1	Indoleamine-2,3-dioxygenase 1
ICG-001	Selective Wnt/β-Catenin Signaling Inhibitor
IGF-1	Insulin-like Growth Factor
IGFR	Insulin-like Growth Factor Receptor
IHC	Immunohistochemistry
IL-6	Interleukin 6
JQ1	Jun Qi 1
JUN	Jun pProto-oncogene, AP-1 Transcription Factor Subunit
KDM5A	Lysine Demethylase 5A
LCN2	Lipocalin 2
MAPK	Mitogen-Activated Protein Kinase
MDR	Multi-Drug Resistance
MEK	Mitogen-Activated Protein Kinase
MET	Mesenchymal–Epithelial Transition Factor
miRNAs	Small Non-Coding MicroRNAs
MMPs	Matrix Metalloproteases
MPEG-PLA	Methoxy Poly(ethylene glycol)-poly(l-lactic acid)
MS023	Human Type I Pprotein Arginine Methyltransferases (Prmts) Inhibitor
NGFR	Nerve Growth Factor Receptor
NPs	Nanoparticles
NPS1034	AXL/MET Inhibitor
NSCLC	Non-Small Cell Lung Cancer
PD-L1	Programmed Death-Ligand 1
PDT	Photo-Dynamic Therapy
PDX	Patient-Derived Xenograft Model
PEG–PCL–PEG, PECE	Poly(ethylene glycol)–poly(e-caprolactone)–poly(ethylene glycol)
pGO	PEGylated Graphene Oxide
PLGA-PEG	Poly(ethylene glycol)-block-poly(lactide-co-glycolide)
RAF	Raf-1 Proto-Oncogene, Serine/threonine Kinase
RAS	Rat Sarcoma
RBP2	Retinol-Binding Protein 2
rGO-AgNPs	Reduced Graphene Oxide-silver Nanoparticles
RNA	Ribonucleic Acid
RNAPII	RNA Polymerase II
RONS	Reactive Oxygen and Nitrogen Species
ROS	Reactive Oxygen Species
RSL3	Inhibitor of GPX4
RTK	Receptor Tyrosine Kinase
siEphA2	siRNA Silencing EphA2
SLPs	Synthetic Lipoproteins
SMAD2	Mothers Against Decapentaplegic Homolog 2
SORA	Sorafenib
SPION	Superparamagnetic Iron Oxide Nanoparticles
TAM	Tumor-Associated Macrophages
TEAD	Transcriptional Enhanced Associate Domain Transcription Factors
TGF-β	Transforming Growth Factor Beta
Thio	Thioridazine
THZ1	CDK7 Inhibitor
Tie2R	Angiopoietin Receptor
TKI	Tyrosine Kinase Inhibitor
TME	Tumor Micro Environment
TNF-α	Tumor Necrosis Factor-α
TSA	Trichostatin A
VEGF	Vascular Endothelial Growth Factor
VEGFR	Vascular Endothelial Growth Factor Receptor
YAP	Yes-associated Protein Transcriptional Coactivator
XL-880	Foretinib
